# Auxin Homeostasis and Distribution of the Auxin Efflux Carrier PIN2 Require Vacuolar NHX-Type Cation/H^+^ Antiporter Activity

**DOI:** 10.3390/plants9101311

**Published:** 2020-10-03

**Authors:** Shiqi Zhang, Hiromi Tajima, Eiji Nambara, Eduardo Blumwald, Elias Bassil

**Affiliations:** 1 Boyce Thompson Institute, Ithaca, NY 14850, USA; shqzhang@ucdavis.edu; 2 Department of Plant Sciences, University of California, Davis, CA 95616, USA; htajima@ucdavis.edu (H.T.); eblumwald@ucdavis.edu (E.B.); 3 Department of Cell and Systems Biology, University of Toronto, Toronto, ON M5S 1A1, Canada; eiji.nambara@utoronto.ca; 4 Horticultural Sciences Department, Tropical Research and Education Center, University of Florida, Homestead, FL 33031, USA

**Keywords:** vacuole, potassium, homeostasis, NHX, auxin distribution, PIN, intracellular trafficking

## Abstract

The *Arabidopsis* vacuolar Na^+^/H^+^ transporters (NHXs) are important regulators of intracellular pH, Na^+^ and K^+^ homeostasis and necessary for normal plant growth, development, and stress acclimation. *Arabidopsis* contains four vacuolar NHX isoforms known as AtNHX1 to AtNHX4. The quadruple knockout *nhx1nhx2nhx3nhx4*, lacking any vacuolar NHX-type antiporter activity, displayed auxin-related phenotypes including loss of apical dominance, reduced root growth, impaired gravitropism and less sensitivity to exogenous IAA and NAA, but not to 2,4-D. In *nhx1nhx2nhx3nhx4,* the abundance of the auxin efflux carrier PIN2, but not PIN1, was drastically reduced at the plasma membrane and was concomitant with an increase in PIN2 labeled intracellular vesicles. Intracellular trafficking to the vacuole was also delayed in the mutant. Measurements of free IAA content and imaging of the auxin sensor DII-Venus, suggest that auxin accumulates in root tips of *nhx1nhx2nhx3nhx4.* Collectively, our results indicate that vacuolar NHX dependent cation/H^+^ antiport activity is needed for proper auxin homeostasis, likely by affecting intracellular trafficking and distribution of the PIN2 efflux carrier.

## 1. Introduction

Plant nutrients are essential to numerous biochemical and physiological processes needed for plant growth and development. Many cellular processes depend on specific steady-state ion concentrations within intracellular compartments. Given the fundamental importance of cellular ion homeostasis, plants contain conserved primary and secondary transport systems and sophisticated mechanisms to regulate their activities to facilitate homeostasis [1]. Primary transporters are H^+^-translocating enzymes, such as H^+^-ATPases on the plasma membrane (PM) and intracellular organelles and vacuolar pyrophosphatase, which use the hydrolysis of ATP and PPi to establish an electrochemical H^+^ gradient across membranes [2]. Secondary transporters use the H^+^ gradient generated by the H^+^-pumps, to drive the uptake of ions and molecules against their electrochemical gradients [1,3,4]. One important group of secondary transporters, known as the NHX-type cation-H^+^ antiporters, mediate the electroneutral exchange of H^+^ for either Na^+^ or K^+^ and, therefore, play key roles in pH regulation, Na^+^ sequestration, and intracellular K^+^ homeostasis. In plants, K^+^ is vital to biosynthesis, osmotic regulation, and charge balance, hence its intracellular levels are tightly regulated [5]. Most plants with known genomes have three orthologous groups of NHX-type antiporters that localize to distinct intracellular compartments. In *Arabidopsis*, four isoforms known as AtNHX1 through AtNHX4 localize to vacuoles [6,7], AtNHX5 and AtNHX6 localize to Golgi, the *trans*-Golgi network (TGN) and pre-vacuolar compartments [8,9,10,11,12], while the two divergent isoforms AtNHX7/SOS1, AtNHX8 localize on the PM [13,14].

Several studies provided compelling evidence to support the role of NHX-type antiporters in the regulation of pH and transport of K^+^ into the vacuole. The *Arabidopsis nhx1nhx2* knockout mutant NHXs displayed acidic pH and 70% less vacuolar K^+^ compared to wild type (WT) plants [6,15]. The *nhx1nhx2* mutant also showed aberrant flower development, reduced vegetative growth, overall smaller cells likely caused by reduced vacuolar K^+^ uptake and insufficient turgor needed to drive cell expansion. Surprisingly, the *nhx1nhx2* mutant also showed severe growth reduction when treated with high K^+^ concentration in the growth media but did not show a similar phenotype in the presence of equimolar Na^+^ concentrations [6]. Moreover, moderate amounts of Na^+^ in the grow media improved its growth. A recent study assessed the contribution of each of the four *Arabidopsis* vacuolar NHXs (NHX1, NHX2, NHX3 and NHX4) to vacuolar Na^+^ and K^+^ transport [16]. Using *nhx* triple and quadruple knockout mutants, the affinity of each vacuolar NHX for Na^+^ and K^+^ and the kinetics of the cation/H^+^ were analyzed [16]. While all four vacuolar NHX antiporters were able to mediate Na^+^ transport, only NHX1, NHX2 and NHX4 but not NHX3 were able to mediate K^+^ transport. Therefore, in the *nhx1/2* or *nhx1/2/3/4* mutant, the loss of major vacuolar K^+^ uptake could lead to an accumulation of K^+^ in the cytosol, as evidenced previously for *nhx1nhx2* [6,15].

It has been suggested that K^+^ transport and cellular K^+^ homeostasis play critical roles in auxin homeostasis. *Arabidopsis* mutants lacking HAK/KUP/KT K^+^ transporters showed defects in root gravitropism, cell expansion and shoot development and abnormal distribution of auxin reporters [17,18,19,20,21,22,23]. Auxin homeostasis is largely regulated by polar auxin transport. In the shoot, auxin flows from the shoot apical meristem, its primary site of biosynthesis, down to the root [24,25] where it moves in two antiparallel streams. Shoot-derived auxin flows down toward the root apex through the central stele, then travels to the outer cells and is redirected to the base of the root [26,27,28]. The downward auxin flow to the root tip (acropetal auxin transport) is mainly associated with an auxin efflux carrier, PIN1(PIN-FORMED 1), whereas the auxin transport from the root tip to the base (basipetal auxin transport) relies on another auxin efflux carrier, PIN2 [29,30]. The PIN family and the ATP-binding cassette (ABC) superfamily of transporters are the main efflux carriers for the polar auxin transport and play important roles in regulating auxin distribution between cells [31].

Little is known about the role of vacuolar ion homeostasis, in auxin homeostasis. Here, using the quadruple knockout *nhx1nhx2nhx3nhx4* lacking vacuolar K^+^/H^+^ exchange activity, we assessed auxin-related phenotypes and the distribution of auxin efflux carriers. Our results strongly suggested that the vacuolar NHXs play an important role in modulating auxin homeostasis, likely by affecting PIN2 distribution.

## 2. Results

### 2.1. The Morphological Phenotype of nhx1nxh2nhx3nhx4 Knockout Mutants

**Figure 1:**
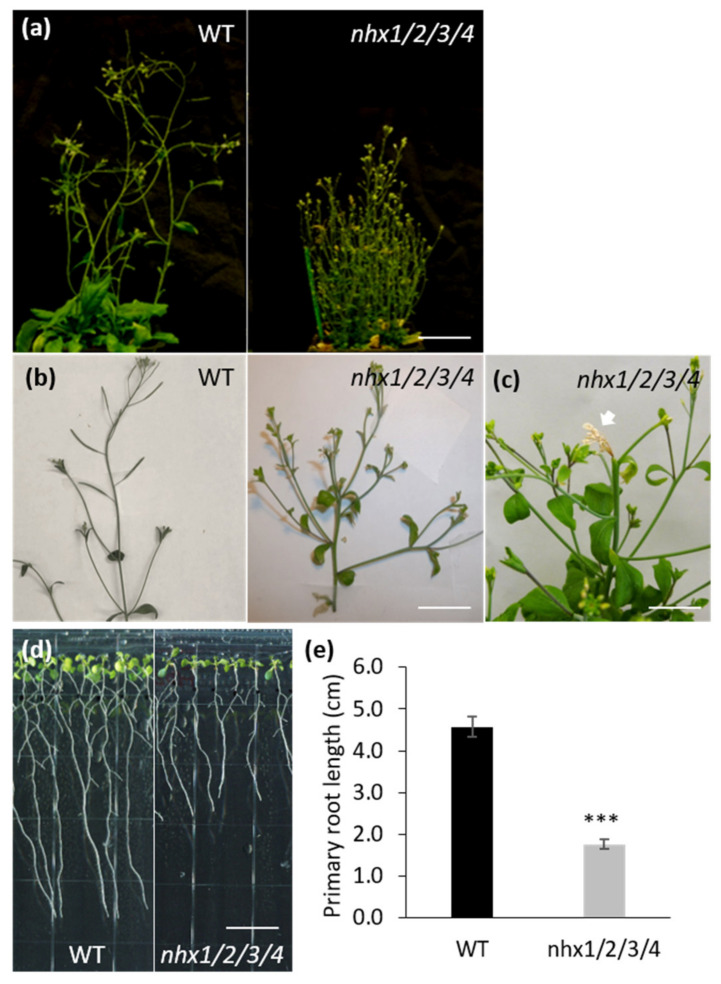
Shoot and root morphological phenotypes of *nhx1nhx2nhx3nhx4* knockout mutant. (**a**), WT (Col-0) and *nhx1/nhx2nhx3nhx4 (nhx1/2/3/4)* were grown in soil under 12-h days for 7 weeks. Scale bar, 4 cm. (**b**), Lateral branches of 4-week-old WT and *nhx1nhx2nhx3nhx4*. Scale bar, 2 cm. (**c**), Shoot apical meristem death of *nhx1nhx2nhx3nhx4* mutants when the plants were 4-week-old. Arrow in (**c**) indicates dead shoot apex and meristem. Scale bar, 0.5 cm. (**d**), WT and *nhx1nhx2nhx3nhx4* grown on standard nutrient plates with 8-h daylight for 7 days. Scale bar, 1 cm. (**e**), Primary root length of WT and the *nhx1nhx2nhx3nhx4* mutant showed in A. Values are mean ± S.D. ***, significant difference (*p* < 0.001; *t* test). n = 50.

The loss of vacuolar NHX activity resulted in plant growth reduction with reduced rosette diameter and decreased growth [16]. When grown under normal growth conditions, the *nhx1nhx2nhx3nhx4* knockout displayed shorter shoots, losses of apical dominance and a profusely ‘bushy’ shoot phenotype (Figure 1a). Shoot branches did not display abnormal phyllotaxy (Figure 1b), suggesting that the knockout did not shift its meristem identity [32]. Close observation of the temporal manifestation of the ‘bushy’ phenotype revealed that the shoot tissue immediately below the inflorescence cluster dies (Figure 1c; arrow) before seed set. This is then followed by the growth of lower second-order shoots, (presumably because they are no longer suppressed by the primary shoot tip). Secondary order shoots exhibit the same shoot tip death as described for the primary shoot and its inflorescences. This is then followed by the growth of third-order shoot buds, and so forth, so that the sequence of shoot tip death and lower order bud outgrowth is repeated until a profusely bushy shoot occurs as shown in Figure 1a. We also examined the root growth and responses to the gravity of the *nhx1nhx2nhx3nhx4* mutant. Mutant roots were much shorter than WT roots grown under the same conditions (Figure 1d). Notably, roots of *nhx1nhx2nhx3nhx4* showed altered gravitropism in which a half of the roots displayed incomplete root bending upon gravistimulation under 1 mM K^+^ and a complete loss of gravitropism in the presence of 30 mM K^+^ (Figure S1). *nhx1nhx2nhx3nhx4* roots grown on 30 mM K^+^ medium also showed aberrant curling away from the gravity vector and is likely to be associated with altered cortical microtubule organization as reported previously [7]. Collectively, the bushy shoot phenotype, the losses of shoot apical dominance and abnormal root gravitropism in the *nhx1nhx2nhx3nhx4* mutant suggest that auxin-associated processes and/or homeostasis could be affected.

### 2.2. The nhx1nhx2nhx3nhx4 Knockout Mutant Exhibited Altered Responses to Auxin

**Figure 2:**
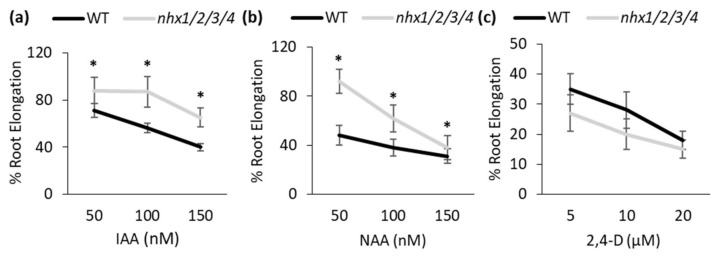
The *nhx1nhx2nhx3nhx4* mutant exhibited less sensitivity to IAA and NAA but not to 2,4-D. (**a**–**c**). Primary root growth of WT and mutant grown on medium supplemented with IAA (**a**), NAA (**b**) and 2,4-D (**c**). Values are the mean ± S.D. (n = 15). Stars indicate significant differences between wild type (WT) and *nhx1nhx2nhx3nhx4 (nhx1/2/3/4)* in the same concentration of auxin. *p* < 0.05 by *t* test.

To evaluate whether the growth phenotypes displayed by the *nhx1nhx2nhx3nhx4* mutant correlated with auxin-associated perturbations, we assessed the root growth responses to supplemental natural auxin (IAA), and the synthetic auxins NAA (1-Naphtaleneacetic acid) and 2,4-D (2,4-Dichlorophen-oxyacetic acid). The transport of IAA is conducted by both auxin influx and efflux systems, however, unlike IAA, 2,4-D transport is favored by auxin influx carriers such as AUX1, but not the auxin efflux carriers PIN2 [33,34]. NAA transport is favored by auxin efflux carriers but not by the influx carriers [35,36,37], as shown by the *aux1* mutant’s resistance to exogenous 2,4-D but not NAA and the *pin2* mutant’s hypersensitivity to exogenous NAA but not 2,4-D. Four-day-old WT and *nhx1nhx2nhx3nhx4* mutant seedlings were transferred to plates containing designated concentrations of IAA, NAA or 2,4-D and grown for 5 days. The root elongation of the primary root was measured and normalized to that of no auxin control (Figure 2). The root elongation of *nhx1nhx2nhx3nhx4* mutant was less sensitive to IAA or NAA compared with that of the WT, whereas the response to 2,4-D is similar to WT seedlings. Altered sensitivity of *nhx1nhx2nhx3nhx4* mutant to IAA and NAA, but not to 2,4-D, suggests that auxin efflux could be affected.

### 2.3. Decreased PIN2 Abundance on the PM of nhx1nhx2nhx3nhx4 Mutant

**Figure 3:**
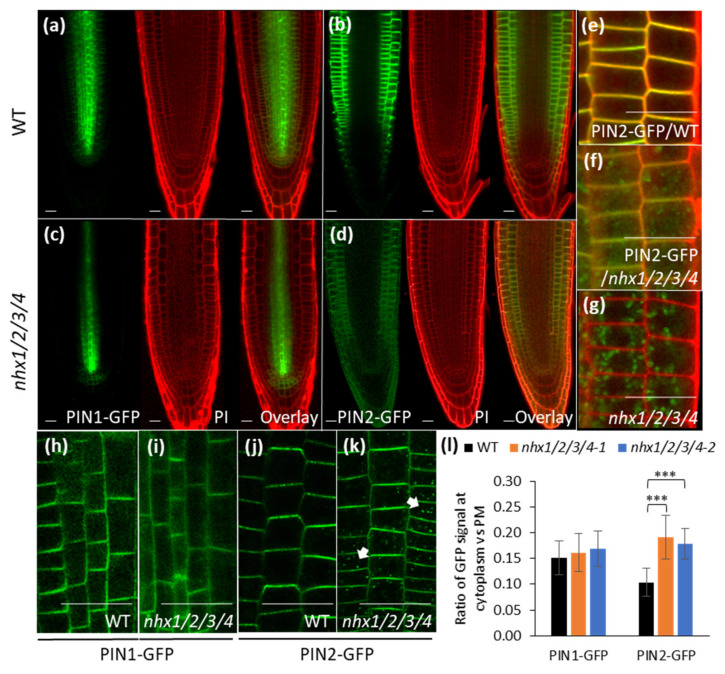
Expression and subcellular localization of PIN1 and PIN2. wild type (WT) and *nhx1nhx2nhx3nhx4* (*nhx1/2/3/4)* root tips are shown in (**a**,**b**) respectively. Localization of PIN1-GFP (**a**) and PIN2-GFP (**b**) in WT; and PIN1-GFP (**c**) and PIN2-GFP (**d**) in *nhx1nhx2nhx3nhx4*. Closeup of PIN2-GFP in WT (**e**) and *nhx1nhx2nhx3nhx4* (**f**). (**g**), autofluorescence signal (green) in *nhx1nhx2nhx3nhx4* roots. Roots were counter stained with propidium iodide (PI) in (**a**–**g**). (**h**,**i**), the subcellular localization of PIN1-GFP in WT (**h**) and *nhx1nhx2nhx3nhx4* (**i**). (**j**,**k**), the subcellular localization of PIN2-GFP in WT (**j**) and *nhx1nhx2nhx3nhx4* (**k**). Arrows in (**k**) indicate the intracellular PIN2-GFP vesicles. (**l**), the ratio of PIN1-GFP and PIN2-GFP signal intensity in the cytosol to that at the PM (values are mean ± SD; *n* ≥ 10; ***, *p* < 0.001 by *t* test). Scale bar, 20 µm.

To assess defects in auxin transport in the *nhx1nhx2nhx3nhx4* mutant, we generated translational fusion reporter lines expressing labelled auxin efflux carriers, PIN1 and PIN2, in the *nhx1nhx2nhx3nhx4* background. Confocal microscopy indicated only a slight difference in the PM-localized signal of PIN1 in the stele cells of the mutants compared to WT (Figure 3a,c) under 1 mM K^+^ condition. The PM-localized PIN2 signal, however, was severely reduced in *nhx1nhx2nhx3nhx4* root cells (Figure 3b,d). Quantitative real-time PCR indicated no difference in the expression of PIN1 and PIN2 transcripts between WT and *nhx1nhx2nhx3nhx4* (Figure S2), suggesting that the reduced PM-localized PIN2 in the mutant was not due to transcriptional regulation. Furthermore, no differences in the subcellular distribution of PIN1 (basal side of stele cells) between WT and *nhx1nhx2nhx3nhx4* were noted (Figure 3h,i,l). However, numerous autofluorescent bodies were seen in *nhx1nhx2nhx3nhx4* root cells (Figure 3e,f,g). These bodies were relatively static and absent from WT roots. To further assess the nature of the PIN2-GFP signal, we used a Leica TCS SP8 confocal microscope equipped with the LightGate function. LightGate is an adjustable temporal window of emission detection that reduces autofluorescence signals. When a pulsed laser beam excites a fluorophore, a strong burst occurs that decays over time, with autofluorescence typically occurring late in the pulse. LightGate can exclude emission from this period thus reducing autofluorescence signals. This particular feature enabled us to adjust the desired imaging window during fluorophore decay to optimize signal collection and to resolve signals emitted from the autofluorescent bodies. Following image optimization, we were able to resolve apparent autofluorescence from an additional signal associated with PIN2-GFP vesicles in the mutant but not WT PIN2-GFP (Arrows in Figure 3j,k). In *nhx1nhx2nhx3nhx4*, PIN2-GFP was also seen on the apical side of the PM as expected but at relatively lower amounts. Quantification of the ratio of PIN2-GFP fluorescence intensity associated with vesicles to that on the PM, was significantly higher in *nhx1nhx2nhx3nhx4* mutant root cells (Figure 3l).

In addition, we conducted a λ (wavelength) scan and linear unmixing of signals obtained from WT PIN2-GFP, *nhx1nhx2nhx3nhx4* PIN2-GFP as well as *nhx1nhx2nhx3nhx4* to further resolve the nature of the signal from auto-fluorescent bodies (Figure S3a). Linear unmixing also enabled the separation of two distinct signals, one that emanated from autofluorescent bodies and another vesicle-like signal only present in *nhx1nhx2nhx3nhx4* expressing PIN2-GFP (Figure S3b). Results from both imaging approaches suggest the existence of bona fide PIN2 containing vesicles in *nhx1nhx2nhx3nhx4* root cells but not WT. Additionally, the subcellular localization of PIN2 in the *nhx1nhx2nhx3nhx4* under 30 mM K^+^ condition were observed because the severe loss of gravitropism occurred under this condition. Surprisingly, no difference in the intracellular PIN2 containing vesicles was found compared to the low K^+^ condition (Figure S4). An accumulation of PIN2-containing vesicles in *nhx1nhx2nhx3nhx4* could result in lower levels of PM-localized PIN2, suggesting defects in the distribution of PIN2.

### 2.4. Intracellular Trafficking to the Vacuole is Affected in the nhx1nhx2nhx3nhx4 Mutant

**Figure 4:**
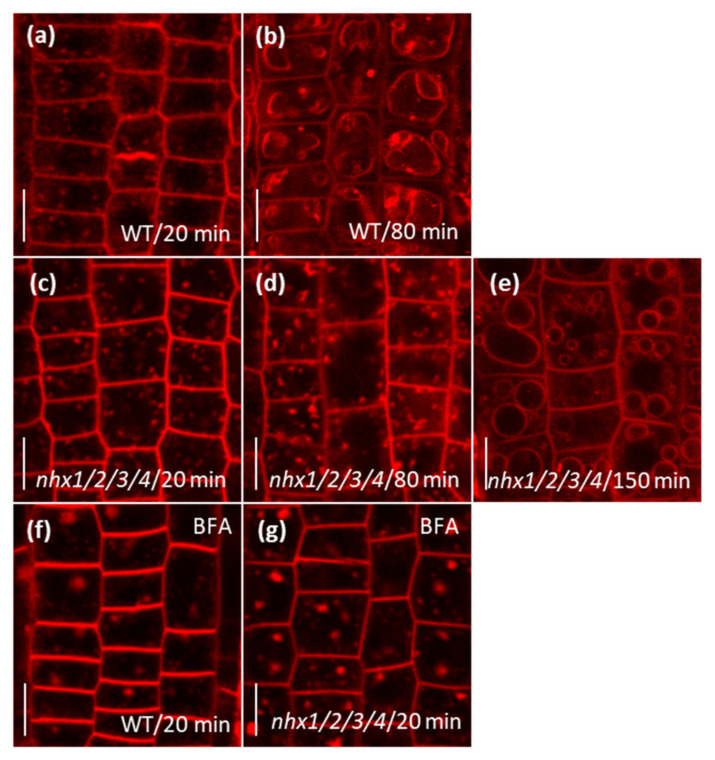
Trafficking to the vacuole is delayed in the *nhx1nhx2nhx3nhx4 mutant.* (**a**,**b**), wild type (WT) root tip cells after 20 min (**a**) or 80 min (**b**) after FM4–64 application. (**c**–**e**), nhx1nhx2nhx3nhx4 (*nhx1/2/3/4)* root tip cells after 20 min (**c**), 80 min (**d**) or 150 min (**e**) after FM4–64 application. (**f**,**g**), formation of BFA bodies in the root tip cells of the WT (**f**) or *nhx1nhx2nhx3nhx4* mutant (**g**). Scale bars, 10 µm.

Given the presence of abundant PIN2-GFP expressing vesicles in *nhx1nhx2nhx3nhx4* and the known constitutive cycling of PIN2 between the PM and endosomal compartments [38,39], we asked whether intracellular trafficking in the mutant might be affected. Because the fluorescence signal of PIN2 is extremely low and not easily discernable without resolving additional autofluorescent signals present in *nhx1nhx2nhx3nhx4* (described above), we used instead the lipophilic dye FM4–64 to observe endomembrane labeling and trafficking in the mutant. In the root tip cells, FM4–64 initially labels the PM and then quickly internalizes into endosomal bodies that traffics and label initially the TGN [40], following other endomembrane compartments including the tonoplast [41]. WT and the mutant roots were stained with 4 µM FM4–64 for 5 min. In both WT and mutant root tips, FM4–64 labeled endosomes became evident after approximately 20 min (Figure 4a,c). As expected, labeling of the vacuolar membrane occurred after approximately 80 min in the WT, but in the mutant, only large FM4–64 endosomal aggregates were noted without any significant labeling of the tonoplast (Figure 4b,d). Instead, vacuolar staining in the mutant occurred after 150 min (Figure 4e). This observation suggested that trafficking from the TGN or pre-vacuolar compartment to the vacuole may be delayed in the *nhx1nhx2nhx3nhx4* mutant.

Given the extensive use of Brefeldin A in assessing endomembrane trafficking processes, we used it to assess whether PIN2 cycling between the PM and endosomal compartments could be affected [39,42] in *nhx1nhx2nhx3nhx4*. Roots were pretreated with 25 µM BFA, then stained with FM4–64 and monitored for the progression of endomembrane labeling. In both WT and *nhx1nhx2nhx3nhx4* root tips pre-treated with BFA, similar-sized BFA bodies were observed after 20 min of FM4–64 application (Figure 4f,g), which suggests that the early internalization of PIN2 and its trafficking to the TGN (which is particularly sensitive to BFA) are not likely to be affected in the *nhx1nhx2nhx3nhx4* mutant.

### 2.5. Auxin Accumulated in the Root Tip Cells of nhx1nhx2nhx3nhx4 Mutant

**Figure 5:**
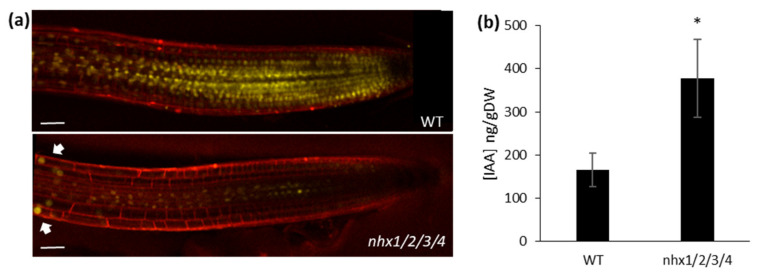
Root cells of *nhx1nhx2nhx3nhx4* accumulate high levels of auxin. (**a**), DII-Venus expression in WT (upper) and *nhx1nhx2nhx3nhx4 (nhx1/2/3/4)*. *nhx2nhx3nhx4 (nhx1/2/3/4)* mutant grown under control conditions. Cells were labeled with propidium iodide (red) as a counter stain to show cell organization. Arrows indicate DII-Venus signals recovery in the elongation zone. Scale bar, 50 µm. (**b**), Free-IAA content measured from the whole root of WT and *nhx1nhx2nhx3nhx4*. Values are the Mean ± SD (n = 3). Star indicates statistical significance (*p* < 0.05 by *t* test).

The decrease in PM-localized PIN2, but not PM-localized PIN1, suggested that the basipetal movement of auxin, but not its acropetal movement, was perturbed in *nhx1nhx2nhx3nhx4* roots. Thus, auxin transported down to the root tip via PIN1 could be accumulated at the root tip. To test this hypothesis, we transformed *nhx1nhx2nhx3nhx4* mutants with the genetically encoded auxin sensor, DII-Venus [43]. DII-Venus is a tandem fusion of a fast-maturing form of YFP fused to the auxin interaction domain, Domain II of Aux/IAA, which is rapidly degraded in response to increases in auxin levels. Therefore, the DII-Venus signal is negatively correlated to the intracellular auxin content., i.e., a decrease in DII-Venus signal intensity indicates an increase in cellular auxin contents. In wild-type plants, the DII-Venus signal was easily detected and abundant (Figure 5a). In *nhx1nhx2nhx3nhx4*, however, DII-Venus decreased drastically in the root tip, meristem, and part of the elongation zone. In cells of the elongation zone, the DII-Venus signal was more similar to WT roots (arrows in Figure 5a), indicating that auxin was accumulating in mutant root tips. To further confirm these observations, we measured the free-IAA content in roots using LC-ESI-MS/MS and found that *nhx1nhx2nhx3nhx4* root tips had double the IAA content compared to wild type root tips (Figure 5b).

## 3. Discussion

The *nhx1nhx2nhx3nhx4* mutant displayed significant growth and developmental phenotypes that could be auxin-related, including the loss of shoot apical dominance, aberrant branching and reduced root growth (Figure 1). We evaluated whether such phenotypes were associated with perturbations in auxin homeostasis, by examining the growth response of the *nhx1nhx2nhx3nhx4* mutant to three auxin isoforms. The *nhx1nhx2nhx3nhx4* mutant showed less sensitivity to IAA and NAA but not to 2,4-D (Figure 2), suggesting that the mutant may be defective in root auxin efflux. Using GFP reporters and confocal microscopy, we examined the distribution of two important auxin efflux carriers, PIN1 and PIN2. Imaging in root tip cells indicated that the PIN1 was similar between the *nhx1nhx2nhx3nhx4* mutant and WT, however, PIN2 abundance at the PM was drastically reduced in *nhx1nhx2nhx3nhx4.* In the mutant, we noted PIN2 labeled vesicles that were observed in WT root cells (Figure 3) suggesting that the cellular distribution of PIN2 could be affected in the mutant. We performed endomembrane labeling experiments using FM-4–64 to monitor intracellular trafficking to the vacuole and whether this was affected in the *nhx1nhx2nhx3nhx4* mutant (Figure 4), and could explain the low abundance of PIN2 at the PM and its presence in intracellular vesicles in *nhx1nhx2nhx3nhx4* roots cells. A lack of PM-localized PIN2 protein could lead to impaired basipetal auxin transport, further resulting in auxin accumulation as evidenced by a low DII-Venus expression and higher free IAA content in mutant root tips (Figure 5).

Vacuolar NHX-type antiporters function coordinately with other primary and secondary transporters to maintain intracellular pH and K^+^ homeostasis [5]. A lack of any vacuolar NHX activity, as shown in the *nhx1nhx2nhx3nhx4* mutant [16], results in acidic vacuoles and little to no vacuolar K^+^ uptake [16]. This could cause aberrant accumulation of K^+^ in the cytosol because of the lack of K^+^ exchange with the vacuole to modulate changes in K^+^ uptake and maintain constant cytosolic K^+^ concentrations [5,6,15,16]. Not much is known about how K^+^ homeostasis could affect intracellular auxin homeostasis but indirect evidence could suggest possible connections. A K^+^ carrier TRH1 (Tiny Root Hair 1) was shown to be involved in auxin transport in *Arabidopsis* roots [21]. Also in *Arabidopsis* roots, ZIFL1.1, one of two alternate splice isoforms of vacuolar-membrane localized ZIFL1 (Zinc-Induced Facilitator-Like 1) displayed H^+^-coupled K^+^ transport activity and was indirectly modulating basipetal auxin efflux likely by regulating the abundance of PIN2 at the PM [44,45]. Other intracellular antiporters of the same family, NHX5 and NHX6 which localize to the TGN, could affect auxin transport by affecting pH of the endoplasmic reticulum [46]. Very recently, Yang et al. demonstrated in rice that a PM H^+^/K^+^ symporter, OsHAK5, indirectly regulates auxin transport by affecting pH [47]. Collectively, such findings, together with our results, highlight the importance of intracellular pH and/or K^+^ homeostasis in maintaining auxin homeostasis.

The molecular mechanism by which vacuolar NHX activity affects the abundance of PIN2 at the PM remains unclear. It is known that the abundance of PIN2 at the PM is maintained by endocytic internalization from the PM and its recycling back to the PM [48,49,50]. During PIN2 cycling, a population of PIN2 containing vesicles is targeted to the vacuole for degradation [39]. It is possible, therefore, that the accumulation of PIN2 vesicles we observed in the *nhx1nhx2nhx3nhx4* mutant, could be a consequence of defective trafficking to the vacuole, vesicle to vacuole fusion or maturation of late endosomes into vacuoles. It is unlikely that the internalization of PIN2 could be a main cause for the aberrant distribution of PIN2 from PM to vesicles because we did not observe an overall effect of endocytosis in the mutant. Notably, compared to PIN2, the subcellular localization and abundance of PIN1 appear to be less affected in the *nhx1nhx2nhx3nhx4* mutant root tips. One reason could be that PIN1 is under different regulation from PIN2 and may be less affected by intracellular ion homeostasis [30,51]. It is also possible that homeostasis in stele cells, where PIN1 is mainly expressed, is less affected by vacuolar NHX activity because the main NHXs involved in vacuolar K^+^ uptake, NHX1, NHX2 and NHX4 [16], are less expressed in these cells [52,53].

## 4. Materials and Methods

### 4.1. Arabidopsis Seeds, Agrobacteria Strains, Growth Conditions and Treatments

Seedlings of the following lines were used in this study: *Arabidopsis thaliana* ecotype Columbia-0 as WT (Col-0); the mutants *nhx1–1/nhx2–1/nhx3–1/nhx4–1* (*nhx1nhx2nhx3nhx4*) and *nhx1–2/nhx2 crispr/nhx3–2/nhx4 crispr* in the Col-0 background; the transgenic lines are *35s::*DII-Venus (DII-Venus) used in [43], (Col-0 seeds and constructs obtained from the ABRC stock center; https://abrc.osu.edu/), *pPIN1::*PIN1-GFP (PIN1-GFP), *pPIN2::*PIN2-GFP (PIN2-GFP) used in [39,54] (Col-0 seeds and constructs kindly provided by Dr. Jiri Friml, IST Austria) in Col-0 and *nhx1–1/nhx2–1/nhx3–1/nhx4–1*. Because homozygous quadruple knockouts produce few seeds, heterozygous NHX1/*nhx1–1/nhx2–1/nhx3–1/nhx4–1* were used for transformation. Transformation of mutant plants was performed by floral dip [55], and the transgenic plants were screened with 20ug/l hygromycin. Multiple independent lines were selected, selfed, and T_2_ and its progenies were used for imaging. All results were confirmed with two independent lines.

Seeds were treated, germinated on modified Murashige and Skoog media (Control) [56] as described previously [6]. Media were supplemented with either KCl or NaCl as indicated in specific experiments. Indole-3-acetic acid (IAA, Sigma), 1-Naphthaleneacetic acid (NAA, Sigma) and 2,4-Dichlorophen-oxyacetic acid (2,4-D, Sigma) were applied in designated concentrations as a supplementation to the solid growth media. 4 µM of FM4–64 or 25µM BFA was applied to 5-day-old seedlings in liquid growth media supplemented with. Procedures were the same as Bassil et al. [8]. At least five biological replicates were performed for each experiment.

### 4.2. Assays for Root Elongation and Root Gravitropism

To assess the effect of auxin on root growth, WT and *nhx1nhx2nhx3nhx4* seedlings were grown on control plates for 5 d and then transferred to a control medium supplemented with different concentrations of IAA, NAA or 2,4-D. Root length was measured 6 or 7 d after transfer. The percentage of root elongation is measured as described in Yamada et al. [57]. To determine the gravitropic response of the roots, seedlings were grown on corresponding media for six days. Then plates were rotated 90° clockwise. After an additional of 5 d, plates were scanned with an Epson Perfection V370 Photo scanner. Image quantification was performed using ImageJ (https://imagej.nih.gov/ij/).

### 4.3. Fluorescence Microscopy

Confocal laser scanning microscopy was performed on a Carl Zeiss confocal microscope LSM 710 (Zeiss Axio Observer Z.1) unless indicated otherwise. DII-Venus was excited at 458 nm, PIN1-GFP and PIN2-GFP at 488nm and FM4–64 at 514 nm. Imaging of PIN1-GFP and PIN2-GFP was also performed on a Leica confocal microscope (Leica TCS SP8 STED 3X) using the LightGate function to reduce autofluorescence of intracellular bodies visible in the GFP emission wavelengths in the *nhx1nhx2nhx3nhx4* knockout. LightGate is an adjustable time window that can turn off emission collection during pulsed white light excitation and which reduces background fluorescence. Linear unmixing was performed using the Zen software of the LSM710 Zeiss confocal microscope. Image quantification was performed using ImageJ (https://imagej.nih.gov/ij/).

### 4.4. IAA Content Measurement

Extraction, purification and measurement of IAA were performed as described in Lu et al. [58] with minor modifications. Briefly, freeze-dried materials were homogenized by TissueLyser (Qiagen), then extracted with methanol containing 1% acetic acid. D2-IAA (Olchemim) was added to the samples prior to methanol extraction. Solid-phase extraction was performed with Oasis HLB cartridge columns. Samples were subjected to liquid chromatography equipped with electrospray ionization tandem mass spectrometry (LC-ESI-MS/MS, Agilent 6410 TripleQuad LC/MS system, Agilent). LC conditions and MS settings are described in Lu et al. [58].

### 4.5. RNA Extraction and qPCR

Transcription of *PIN1* and *PIN2* was examined for WT and *nhx1nhx2nhx3nhx4* genotypes by qPCR. RNA from the roots, 50 seedlings per sample, was extracted using the Qiagen RNase kit. cDNA was reverse-transcribed with the Qiagen Quanti-tect kit. The *Arabidopsis* TIP41-like gene (GenBank accession No. At4g34270.1) served as the reference gene. qPCR was amplified using the specific primers 5′- GCCAGCTCTTATAGCAAAGTC-3′ (forward) and 5′- GGTCCAACGACAAATCTCATAG-3′ (reverse) for *PIN1* and 5′- CTGGTCTTGGAATG-GCTA-3′ (forward) and 5′- AGGAGATCACCTCGAATA-3′ (reverse) for *PIN2*. qPCR amplification was performed as described by [6] using the 2−ΔΔC_T_ method [59]. The experiment was performed using three biological replicates, with two technical repeats for each. Means ± standard deviations (SD) were calculated for all biological replicates. Means of replicates were subjected to statistical analysis by a Student’s t-test (*P* ≤ 0.05) using Prism 6.

## 5. Conclusions

This study provides evidence to link pH and/or K^+^ homeostasis to auxin homeostasis. Using a quadruple *nhx1nhx2nhx3nhx4* mutant which lacks vacuolar NHX-type antiport activity that is severely affected in vacuolar pH and K^+^ uptake, we examined auxin related phenotypes and growth responses. Our data suggest that auxin efflux is disrupted. Imaging of the abundance and localization of the auxin efflux carriers PIN1 and PIN2 in the mutant, showed that PM-localized PIN2 abundance, but not PIN1, was drastically reduced and was concomitant with an increase in PIN2 labelled intracellular vesicles. The *nhx1nhx2nhx3nhx4* mutant also showed delayed intracellular trafficking to the vacuole and auxin accumulation in the root tip. Collectively, these results indicate that the vacuolar NHXs are required for proper cellular and tissue auxin homeostasis, likely by affecting the intracellular trafficking and abundance of PIN2 on the plasma membrane.

## Supplementary Materials

The following are available online at https://www.mdpi.com/2223-7747/9/10/1311/s1, Figure S1: Root response to gravistimulation of *nhx1nhx2nhx3nhx4* mutant, Figure S2: The expression level of *PIN1* and *PIN2* genes in WT and *nhx1nhx2nhx3nhx4* mutant roots, Figure S3: Alternate method (linear unmixing) used to eliminate the plastid-like bodies in PIN2-GFP/*nhx1nhx2nhx3nhx4*, Figure S4: Subcellular localization of PIN2 in *nhx1nhx2nhx3nhx4* under 1mM K^+^ and 30mM K^+^.

## References

[1] Blumwald E., Rea P.A., Poole R.J. (1987). Preparation of Tonoplast Vesicles: Applications to H+-Coupled Secondary Transport in Plant Vacuoles, In Methods in Enzymology.

[2] Orlowski J., Grinstein S. (2007). Emerging roles of alkali cation/proton exchangers in organellar homeostasis. Curr. Opin. Cell Biol..

[3] Martinoia E., Maeshima M., Neuhaus H.E. (2007). Vacuolar transporters and their essential role in plant metabolism. J. Exp. Bot..

[4] Amtmann A., Leigh R. (2009). Ion homeostasis. Abiotic Stress Adaptation in Plants.

[5] Bassil E., Coku A., Blumwald E. (2012). Cellular ion homeostasis: Emerging roles of intracellular NHX Na^+^/H^+^ antiporters in plant growth and development. J. Exp. Bot..

[6] Bassil E., Tajima H., Liang Y.C., Ohto M., Ushijima K., Nakano R., Esumi T., Coku A., Belmonte M., Blumwald E. (2011). The Arabidopsis Na^+^/H^+^ Antiporters NHX1 and NHX2 Control Vacuolar pH and K^+^ Homeostasis to Regulate Growth, Flower Development, and Reproduction. Plant Cell.

[7] McCubbin T., Bassil E., Zhang S., Blumwald E. (2014). Vacuolar Na^+^/H^+^ NHX-Type Antiporters Are Required for Cellular K^+^ Homeostasis, Microtubule Organization and Directional Root Growth. Plants.

[8] Bassil E., Ohto M.A., Esumi T., Tajima H., Zhu Z., Cagnac O., Belmonte M., Peleg Z., Yamaguchi T., Blumwald E. (2011). The Arabidopsis Intracellular Na^+^/H^+^ Antiporters NHX5 and NHX6 Are Endosome Associated and Necessary for Plant Growth and Development. Plant Cell.

[9] Martinière A., Bassil E., Jublanc E., Alcon C., Reguera M., Sentenac H., Blumwald E., Paris N. (2013). In Vivo Intracellular pH Measurements in Tobacco and Arabidopsis Reveal an Unexpected pH Gradient in the Endomembrane System. Plant Cell.

[10] Reguera M., Bassil E., Tajima H., Wimmer M., Chanoca A., Otegui M.S., Paris N., Blumwald E. (2015). pH Regulation by NHX-Type Antiporters Is Required for Receptor-Mediated Protein Trafficking to the Vacuole in Arabidopsis. Plant Cell.

[11] Dragwidge J.M., Ford B.A., Ashnest J.R., Das P., Gendall A.R. (2018). Two Endosomal NHX-Type Na^+^/H^+^ Antiporters are Involved in Auxin-Mediated Development in Arabidopsis thaliana. Plant Cell Physiol..

[12] Wu X.X., Ebine K., Ueda T., Qiu Q.S. (2016). AtNHX5 and AtNHX6 Are Required for the Subcellular Localization of the SNARE Complex That Mediates the Trafficking of Seed Storage Proteins in Arabidopsis. PLoS ONE.

[13] Shi H.Z., Quintero F.J., Pardo J.M., Zhu J.K. (2002). The putative plasma membrane Na^+^/H^+^ antiporter SOS1 controls long-distance Na^+^ transport in plants. Plant Cell.

[14] An R., Chen Q.J., Chai M.F., Lu P.L., Su Z., Qin Z.X., Chen J., Wang X.C. (2007). AtNHX8, a member of the monovalent cation: Proton antiporter-1 family in Arabidopsis thaliana, encodes a putative Li^+^/H^+^ antiporter. Plant J..

[15] Barragán V., Leidi E.O., Andrés Z., Rubio L., De Luca A., Fernández J.A., Cubero B., Pardo J.M. (2012). Ion Exchangers NHX1 and NHX2 Mediate Active Potassium Uptake into Vacuoles to Regulate Cell Turgor and Stomatal Function in Arabidopsis. Plant Cell.

[16] Bassil E., Zhang S., Gong H., Tajima H., Blumwald E. (2019). Cation specificity of vacuolar NHX-type cation/H^+^ antiporters. Plant Physiol..

[17] Rigas S., Debrosses G., Haralampidis K., Vicente-Agullo F., Feldmann K.A., Grabov A., Dolan L., Hatzopoulos P. (2001). TRH1 encodes a potassium transporter required for tip growth in Arabidopsis root hairs. Plant Cell.

[18] Rigas S., Ditengou F.A., Ljung K., Daras G., Tietz O., Palme K., Hatzopoulos P. (2013). Root gravitropism and root hair development constitute coupled developmental responses regulated by auxin homeostasis in the Arabidopsis root apex. New Phytol..

[19] Elumalai R.P., Nagpal P., Reed J.W. (2002). A mutation in the Arabidopsis KT2/KUP2 potassium transporter gene affects shoot cell expansion. Plant Cell.

[20] Desbrosses G., Josefsson C., Rigas S., Hatzopoulos P., Dolan L. (2003). AKT1 and TRH1 are required during root hair elongation in Arabidopsis. J. Exp. Bot..

[21] Vicente-Agullo F., Rigas S., Desbrosses G., Dolan L., Hatzopoulos P., Grabov A. (2004). Potassium carrier TRH1 is required for auxin transport in Arabidopsis roots. Plant J..

[22] Osakabe Y., Arinaga N., Umezawa T., Katsura S., Nagamachi K., Tanaka H., Ohiraki H., Yamada K., Seo S.-U., Abo M. (2013). Osmotic stress responses and plant growth controlled by potassium transporters in Arabidopsis. Plant Cell.

[23] Daras G., Rigas S., Tsitsekian D., Iacovides T.A., Hatzopoulos P. (2015). Potassium transporter TRH1 subunits assemble regulating root-hair elongation autonomously from the cell fate determination pathway. Plant Sci..

[24] Okada K., Ueda J., Komaki M.K., Bell C.J., Shimura Y. (1991). Requirement of the auxin polar transport system in early stages of Arabidopsis floral bud formation. Plant Cell.

[25] Rashotte A.M., Poupart J., Waddell C.S., Muday G.K. (2003). Transport of the two natural auxins, indole-3-butyric acid and indole-3-acetic acid, in Arabidopsis. Plant Physiol..

[26] Mitchell E., Davies P. (1975). Evidence for three different systems of movement of indoleacetic acid in intact roots of Phaseolus coccineus. Physiol. Plant..

[27] Tsurumi S., Ohwaki Y. (1978). Transport of 14C-lableled indoleacetic acid in Vicia root segments. Plant Cell Physiol..

[28] Rashotte A.M., Brady S.R., Reed R.C., Ante S.J., Muday G.K. (2000). Basipetal auxin transport is required for gravitropism in roots of Arabidopsis. Plant Physiol..

[29] Křeček P., Skůpa P., Libus J., Naramoto S., Tejos R., Friml J., Zažímalová E. (2009). The PIN-FORMED (PIN) protein family of auxin transporters. Genome Biol..

[30] Adamowski M., Friml J. (2015). PIN-Dependent Auxin Transport: Action, Regulation, and Evolution. Plant Cell.

[31] Zazímalová E., Murphy A.S., Yang H., Hoyerová K., Hosek P. (2010). Auxin transporters—Why so many?. Cold Spring Harb. Perspect. Biol..

[32] Bartlett M.E., Thompson B. (2014). Meristem identity and phyllotaxis in inflorescence development. Front. Plant Sci..

[33] Delbarre A., Muller P., Imhoff V., Guern J. (1996). Comparison of mechanisms controlling uptake and accumulation of 2,4-dichlorophenoxy acetic acid, naphthalene-1-acetic acid, and indole-3-acetic acid in suspension-cultured tobacco cells. Planta.

[34] Marchant A., Kargul J., May S.T., Muller P., Delbarre A., Perrot-Rechenmann C., Bennett M.J. (1999). AUX1 regulates root gravitropism in Arabidopsis by facilitating auxin uptake within root apical tissues. EMBO J..

[35] Chen R.J., Hilson P., Sedbrook J., Rosen E., Caspar T., Masson P.H. (1998). The Arabidopsis thaliana AGRAVITROPIC 1 gene encodes a component of the polar-auxin-transport efflux carrier. Proc. Natl. Acad. Sci. USA.

[36] Muller A., Guan C.H., Galweiler L., Tanzler P., Huijser P., Marchant A., Parry G., Bennett M., Wisman E., Palme K. (1998). AtPIN2 defines a locus of Arabidopsis for root gravitropism control. EMBO J..

[37] Luschnig C., Gaxiola R.A., Grisafi P., Fink G.R. (1998). EIR1, a root-specific protein involved in auxin transport, is required for gravitropism in Arabidopsis thaliana. Genes Dev..

[38] Abas L., Benjamins R., Malenica N., Paciorek T., Wirniewska J., Moulinier-Anzola J.C., Sieberer T., Friml J., Luschnig C. (2006). Intracellular trafficking and proteolysis of the Arabidopsis auxin-efflux facilitator PIN2 are involved in root gravitropism. Nat. Cell Biol..

[39] Kleine-Vehn J., Leitner J., Zwiewka M., Sauer M., Abas L., Luschnig C., Friml J. (2008). Differential degradation of PIN2 auxin efflux carrier by retromer-dependent vacuolar targeting. Proc. Natl. Acad. Sci. USA.

[40] Dettmer J., Hong-Hermesdorf A., Stierhof Y.D., Schumacher K. (2006). Vacuolar H^+^-ATPase activity is required for Endocytic and secretory trafficking in Arabidopsis. Plant Cell.

[41] Bolte S., Talbot C., Boutte Y., Catrice O., Read N.D., Satiat-Jeunemaitre B. (2004). FM-dyes as experimental probes for dissecting vesicle trafficking in living plant cells. J. Microsc..

[42] Kleine-Vehn J., Langowski L., Wisniewska J., Dhonukshe P., Brewer P.B., Friml J. (2008). Cellular and Molecular Requirements for Polar PIN Targeting and Transcytosis in Plants. Mol. Plant.

[43] Brunoud G., Wells D.M., Oliva M., Larrieu A., Mirabet V., Burrow A.H., Beeckman T., Kepinski S., Traas J., Bennett M.J. (2012). A novel sensor to map auxin response and distribution at high spatio-temporal resolution. Nature.

[44] Remy E., Baster P., Friml J., Duque P. (2013). ZIFL1.1 transporter modulates polar auxin transport by stabilizing membrane abundance of multiple PINs inArabidopsisroot tip. Plant Signal. Behav..

[45] Remy E., Cabrito T.R., Baster P., Batista R.A., Teixeira M.C., Friml J., Sá-Correia I., Duque P. (2013). A major facilitator superfamily transporter plays a dual role in polar auxin transport and drought stress tolerance in Arabidopsis. Plant Cell.

[46] Fan L. (2018). Na^+^, K^+^/H^+^ antiporters regulate the pH of endoplasmic reticulum and auxin-mediated development. Plant Cell Environ..

[47] Yang T., Zhao L., Hu H., Li W., Novák O., Strnad M., Simon S., Friml J., Shen J., Jiang L. (2020). The Potassium Transporter OsHAK5 Alters Rice Architecture via ATP-Dependent Transmembrane Auxin Fluxes. Plant Commun..

[48] Geldner N., Friml J., Stierhof Y.-D., Jürgens G., Palme K. (2001). Auxin transport inhibitors block PIN1 cycling and vesicle trafficking. Nature.

[49] Dhonukshe P., Aniento F., Hwang I., Robinson D.G., Mravec J., Stierhof Y.-D., Friml J. (2007). Clathrin-mediated constitutive endocytosis of PIN auxin efflux carriers in Arabidopsis. Current Biol..

[50] Kleine-Vehn J., Dhonukshe P., Sauer M., Brewer P.B., Wiśniewska J., Paciorek T., Benková E., Friml J. (2008). ARF GEF-dependent transcytosis and polar delivery of PIN auxin carriers in Arabidopsis. Current Biol..

[51] Li K., Kamiya T., Fujiwara T. (2015). Differential Roles of PIN1 and PIN2 in Root Meristem Maintenance Under Low-B Conditions in Arabidopsis thaliana. Plant Cell Physiol..

[52] Birnbaum K., Shasha D.E., Wang J.Y., Jung J.W., Lambert G.M., Galbraith D.W., Benfey P.N. (2003). A Gene Expression Map of the *Arabidopsis* Root. Science.

[53] Nawy T., Lee J.-Y., Colinas J., Wang J.Y., Thongrod S.C., Malamy J.E., Birnbaum K., Benfey P.N. (2005). Transcriptional Profile of the Arabidopsis Root Quiescent Center. Plant Cell.

[54] Benkova E., Michniewicz M., Sauer M., Teichmann T., Seifertova D., Jurgens G., Friml J. (2003). Local, efflux-dependent auxin gradients as a common module for plant organ formation. Cell.

[55] Clough S.J., Bent A.F. (1998). Floral dip: A simplified method for Agrobacterium-mediated transformation of Arabidopsis thaliana. Plant J..

[56] Spalding E.P., Hirsch R.E., Lewis D.R., Qi Z., Sussman M.R., Lewis B.D. (1999). Potassium uptake supporting plant growth in the absence of AKT1 channel activity—Inhibition by ammonium and stimulation by sodium. J. Gen. Physiol..

[57] Yamada M., Greenham K., Prigge M.J., Jensen P.J., Estelle M. (2009). The TRANSPORT INHIBITOR RESPONSE2 Gene Is Required for Auxin Synthesis and Diverse Aspects of Plant Development. Plant Physiol..

[58] Lu G., Coneva V., Casaretto J.A., Ying S., Mahmood K., Liu F., Nambara E., Bi Y.M., Rothstein S.J. (2015). OsPIN5b modulates rice (Oryza sativa) plant architecture and yield by changing auxin homeostasis, transport and distribution. Plant J..

[59] Schmittgen T.D., Livak K.J. (2008). Analyzing real-time PCR data by the comparative CT method. Nat. Protoc..

