# Supplementary material for: Auxin Homeostasis and Distribution of the Auxin Efflux Carrier PIN2 Require Vacuolar NHX-Type Cation/H+ Antiporter Activity
Source: Plants (Basel). 2020 Oct 3;9(10):1311. doi: 10.3390/plants9101311 (PMC7601841; doi:10.3390/plants9101311)
Supplement: Supplementary file 1 [file plants-09-01311-s001.zip › Supplemental materal.pptx]

## Slide 1
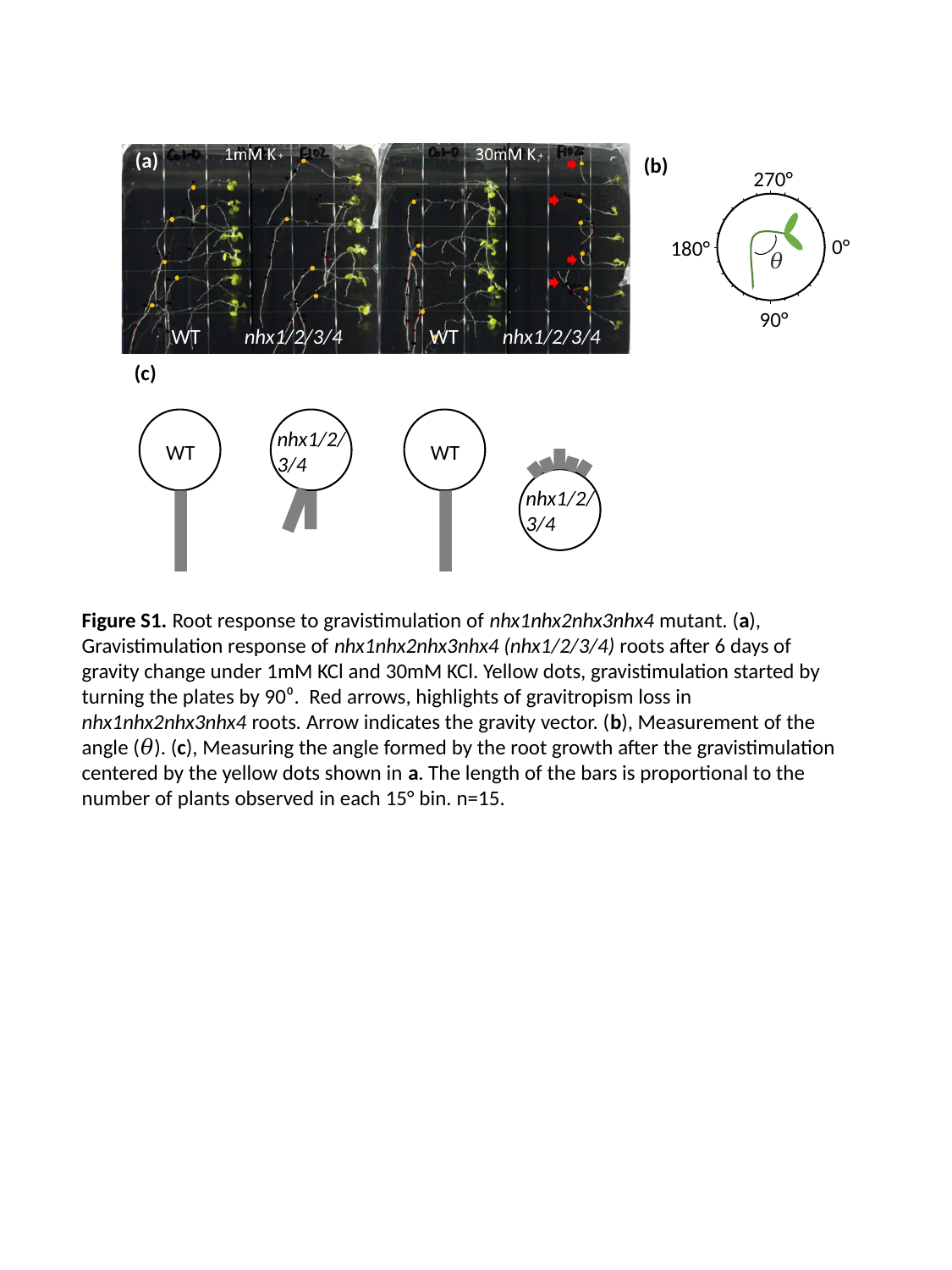

WT nhx1/2/3/4
(a)
+
+
(b)
270°
0°
180°
𝜃
90°
 WT nhx1/2/3/4
 WT nhx1/2/3/4
(c)
+
nhx1/2/
3/4
WT
WT
nhx1/2/
3/4
Figure S1. Root response to gravistimulation of nhx1nhx2nhx3nhx4 mutant. (a), Gravistimulation response of nhx1nhx2nhx3nhx4 (nhx1/2/3/4) roots after 6 days of gravity change under 1mM KCl and 30mM KCl. Yellow dots, gravistimulation started by turning the plates by 90⁰. Red arrows, highlights of gravitropism loss in nhx1nhx2nhx3nhx4 roots. Arrow indicates the gravity vector. (b), Measurement of the angle (𝜃). (c), Measuring the angle formed by the root growth after the gravistimulation centered by the yellow dots shown in a. The length of the bars is proportional to the number of plants observed in each 15° bin. n=15.

## Slide 2
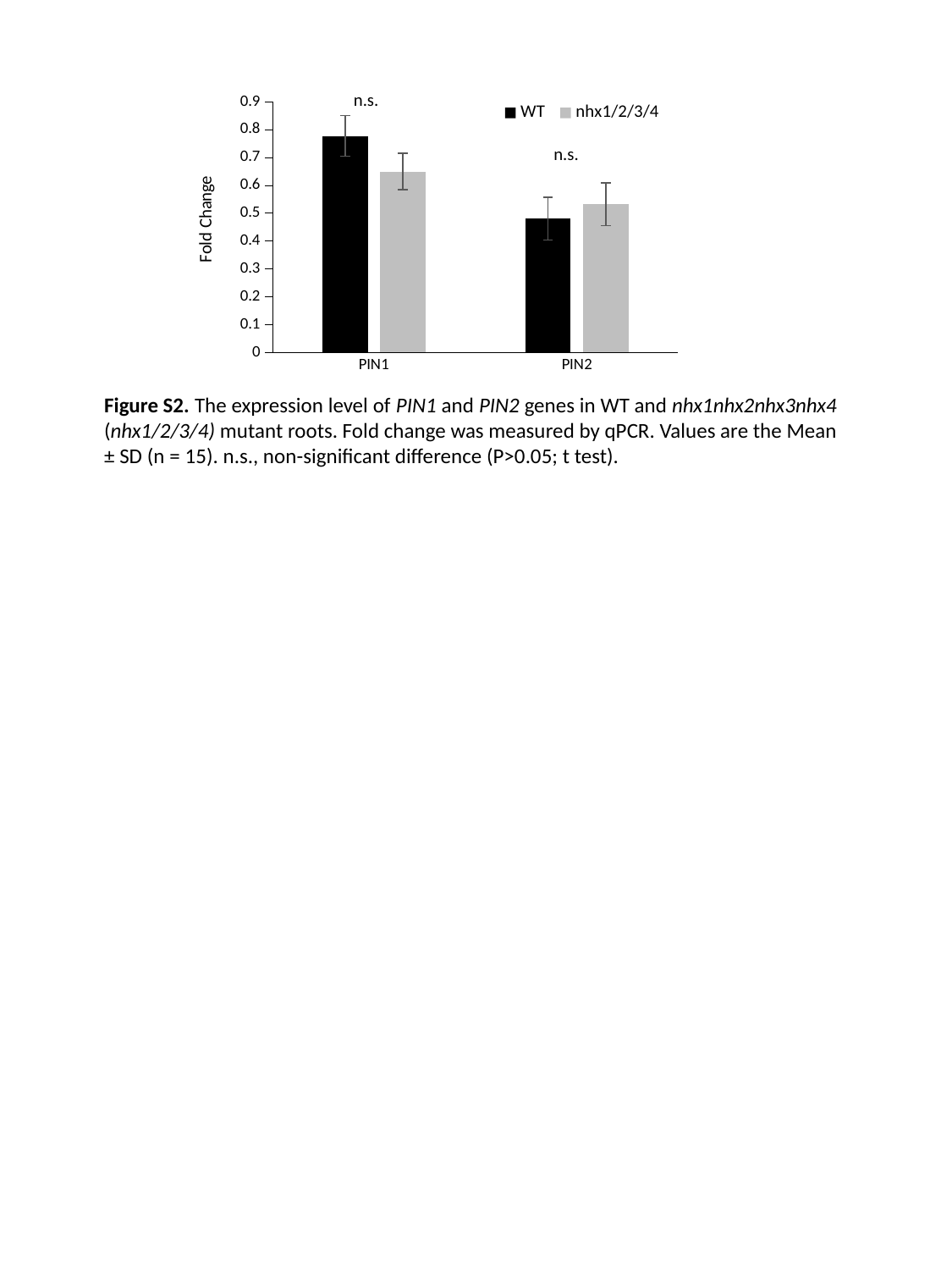

n.s.
### Chart
| Category | | |
|---|---|---|
| | 0.777894123024049 | 0.650169228435891 |
| | 0.480520650242585 | 0.532321132703342 |n.s.
Figure S2. The expression level of PIN1 and PIN2 genes in WT and nhx1nhx2nhx3nhx4 (nhx1/2/3/4) mutant roots. Fold change was measured by qPCR. Values are the Mean ± SD (n = 15). n.s., non-significant difference (P>0.05; t test).

## Slide 3
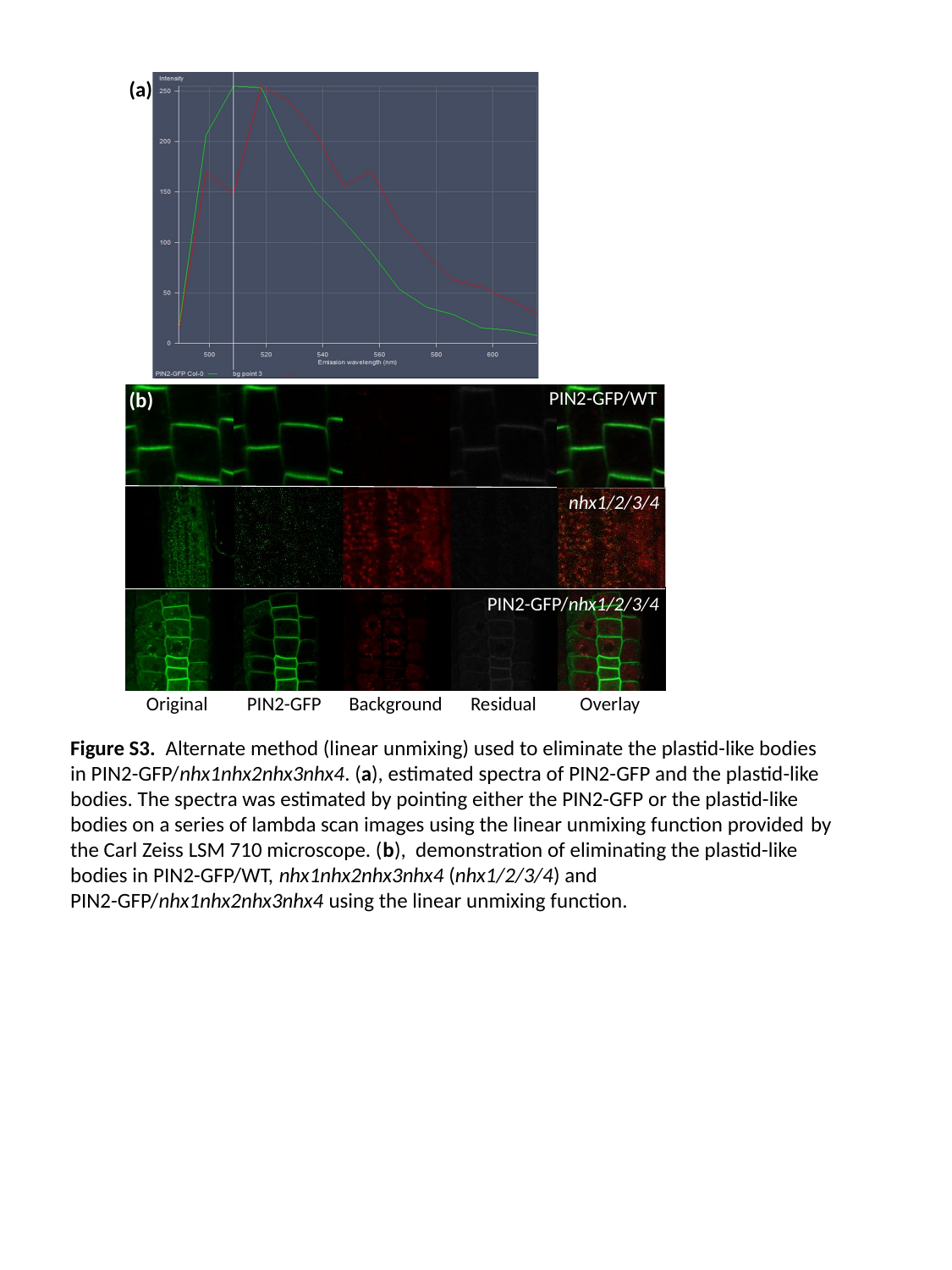

(a)
PIN2-GFP/WT
(b)
nhx1/2/3/4
PIN2-GFP/nhx1/2/3/4
Original
PIN2-GFP
Background
Residual
Overlay
Figure S3. Alternate method (linear unmixing) used to eliminate the plastid-like bodies in PIN2-GFP/nhx1nhx2nhx3nhx4. (a), estimated spectra of PIN2-GFP and the plastid-like bodies. The spectra was estimated by pointing either the PIN2-GFP or the plastid-like bodies on a series of lambda scan images using the linear unmixing function provided by the Carl Zeiss LSM 710 microscope. (b), demonstration of eliminating the plastid-like bodies in PIN2-GFP/WT, nhx1nhx2nhx3nhx4 (nhx1/2/3/4) and PIN2-GFP/nhx1nhx2nhx3nhx4 using the linear unmixing function.

## Slide 4
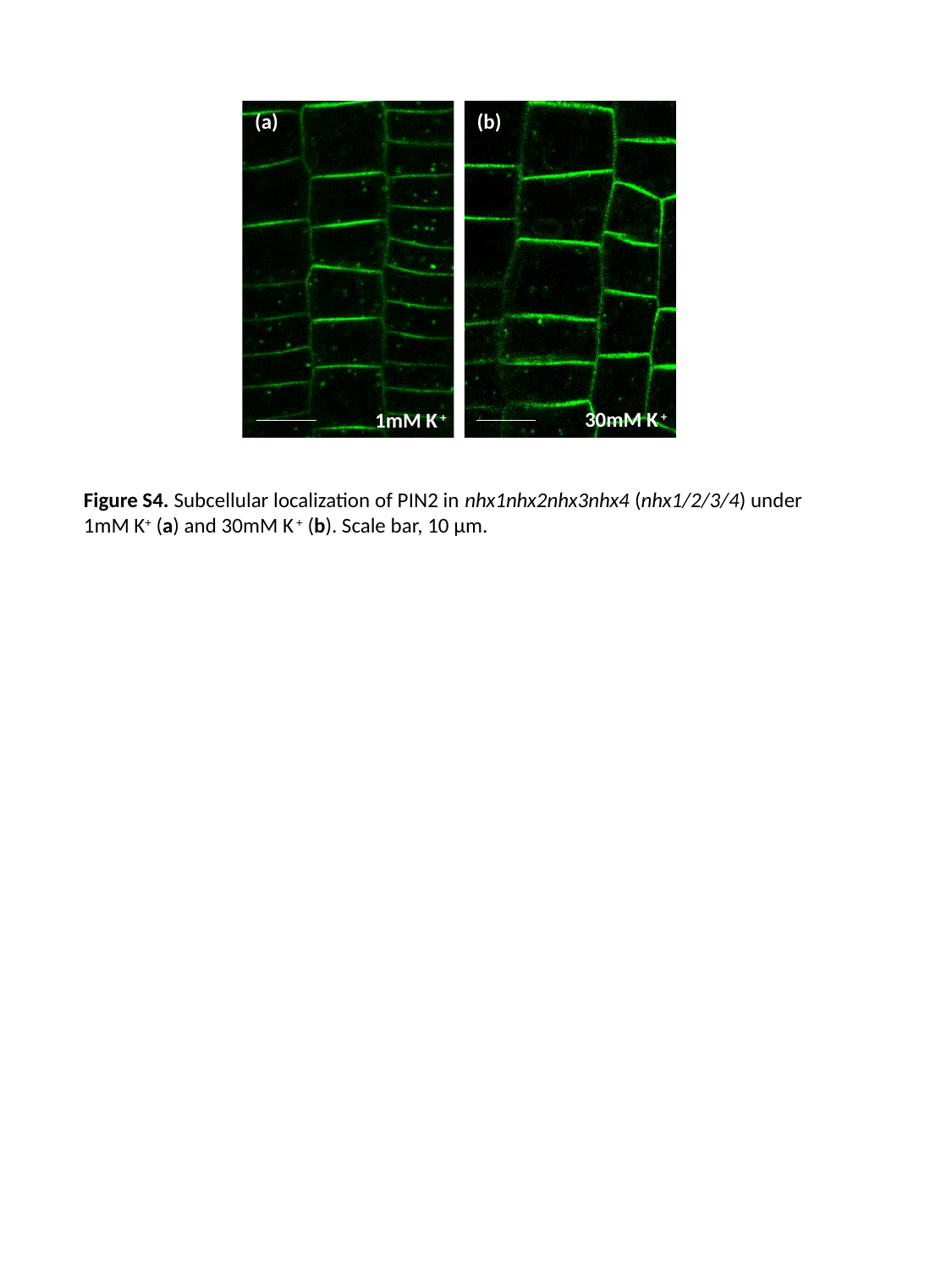

(b)
(a)
30mM K +
1mM K +
Figure S4. Subcellular localization of PIN2 in nhx1nhx2nhx3nhx4 (nhx1/2/3/4) under 1mM K+ (a) and 30mM K + (b). Scale bar, 10 µm.
